# The Use of Bacteriophages in the Poultry Industry

**DOI:** 10.3390/ani10050872

**Published:** 2020-05-18

**Authors:** Katarzyna Żbikowska, Monika Michalczuk, Beata Dolka

**Affiliations:** 1 Department of Animal Breeding, Institute of Animal Sciences, Warsaw University of Life Sciences—SGGW, Ciszewskiego 8 St., 02-786 Warsaw, Poland; katarzyna.zbikowska@fermydrobiubebnowo.pl (K.Ż.); monika_michalczuk@sggw.edu.pl (M.M.); 2 Department of Pathology and Veterinary Diagnostics, Institute of Veterinary Medicine, Warsaw University of Life Sciences—SGGW, Nowoursynowska 159c St., 02-776 Warsaw, Poland

**Keywords:** bacteriophages, poultry, phage therapy, food safety, disinfection, multidrug-resistant bacteria, foodborne diseases

## Abstract

**Simple Summary:**

Poultry production is one of the worldwide sectors which utilizes many antibiotics. Reducing antibiotic use is one of the biggest challenges to the poultry industry globally. Due to the increasing risk of antibiotic-resistant bacteria, the European Union has in 2006 imposed a ban on the use of antibiotics as growth promoters in food-producing animals, but they are still used in other parts of the world. Following the ban, many countries reported a negative impact on animals’ well-being, the re-emergence of old infectious diseases in poultry, and an increase in the usage of antibiotics in poultry for therapeutic purposes. Nowadays, foodborne bacterial pathogens have been considered as the leading bacterial causes of human diseases. In the era of the increasing emergence of multidrug-resistant bacteria and a lack of new effective antibiotics, it is natural that much scientific effort has been put into developing and implementing new technologies to combat bacteria. In this context, bacteriophages (phages) have been proposed as an alternative strategy to antibiotics for poultry, and thus for food safety and public health.

**Abstract:**

The emergence of multidrug-resistant infections and antibiotic failures have raised concerns over human and veterinary medicine worldwide. Poultry production has had to confront the problems of an alarming increase in bacterial resistance, including zoonotic pathogens. According to the European Food Safety Authority (EFSA), campylobacteriosis and salmonellosis have been the most frequently reported human foodborne diseases linked to poultry. This situation has strongly stimulated a renewal of scientists’ interest in bacteriophages (phages) since the beginning of the 21st century. Bacteriophages are the viruses of bacteria. They are abundant in nature, and accompany bacteria in each environment they colonize, including human microbiota. In this review, we focused on the use of bacteriophages as therapeutic agents to treat infections and reduce counts of pathogenic bacteria in poultry, as biocontrol agents to eliminate foodborne pathogens on/in food, and also as disinfectants to reduce contamination on food-contact surfaces or poultry carcasses in industrial conditions. Most of the phage-based products are targeted against the main foodborne pathogens, such as *Campylobacter jejuni*, *Salmonella* spp., *Escherichia coli*, *Listeria monocytogenes*, *Staphylococcus aureus,* and *Clostridium perfringens*. Phages are currently addressed at all stages of the poultry production "from farm to fork", however, their implementation into live birds and food products still provokes discussions especially in the context of the current legal framework, limitations, as well as public health and safety.

## 1. Introduction

Bacteriophages (phages, BPs) are viruses that specifically target and infect prokaryotes (bacteria) or arachea. Bacteriophages were discovered independently by two scientists: Frederick Twort (in 1915) and Felix d’Herelle (in 1917) [1,2,3]. They are globally ubiquitous, with an estimated total number of phage particles in the biosphere approximating 10^31^, which is 10-times more than the estimated number of bacterial cells on the Earth [4]. Bacteriophages are considered as being non-pathogenic to humans. They can be found in all habitats colonized by bacteria, including water, plants, and food, and therefore are frequently consumed by people. Moreover, phages have been recognized as important components of the natural microbiome of humans. The human gut virome (the whole viral community in gut) is predominated by bacteriophage community (known as the phageome). However, little is known about the healthy chicken gut phageome still [5].

Phages are categorized based on their morphology (three-dimensional shaped, pleomorphic, polyhedral, icosahedral, filamentous, head-tail or thread-like shaped), nucleic acid (+ ssRNA, dsRNA, ssDNA, dsDNA), phage life cycle, bacterial target and site. Over 5000 phages have been examined by the electron microscopy, and most of them (96%) had tails. Bacteriophages are classified into many orders and 15 families. The tailed phages constitute the order *Caudovirales,* which is divided into families: *Siphoviridae* (61% of tailed phages), *Myoviridae* (25%), and *Podoviridae* (14%) families [6]. The vast majority of phages belong to the order *Caudovirales* and have a broad range of isometric heads varying from 20 to 200 nm [7] which makes them 1000 times smaller than the average bacterium (0.5–20 µm).

**Figure 1:**
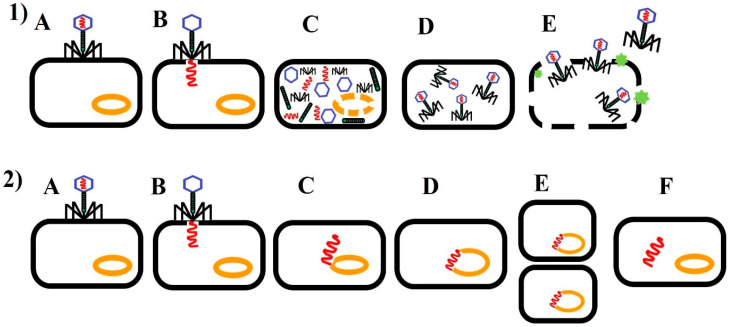
Life cycle of bacteriophage. (1) *Lytic cycle.* (**A**) Attachment: the phage binds to a receptor on the bacterial cell surface. (**B**) Penetration: the phage inserts its DNA into the bacterial cytoplasm. (**C**) DNA replication and protein synthesis: the phage takes over the bacterial cell functions and directs the synthesis to produce of phage DNA copies and proteins. Bacterial DNA is degraded. (**D**) Assembly packaging: new phage particles are assembled within host cell. (**E**) Lysis: the phage produces an enzyme which destroys the bacterial cell wall, causing lysis and the release new phages. The bacterial host cell is destroyed. Progeny phages can infect further bacterial cells and the cycle starts again. (2) *Lysogenic cycle.* (**A**) Attachment: the phage binds to a receptor on the bacterial cell surface. (**B**) Penetration: the entry of phage nucleic acid. (**C**) Integration of phage DNA: the phage DNA then moves through the cytoplasm to the host bacterial DNA and integrates itself into the host genome. (**D**) Prophage stage: the phage DNA is incorporated into the bacterial genome and becomes a (noninfective) prophage. (**E**) The prophage is replicated along with the bacterial genome. The bacterial cell divides and prophage DNA is transferred into daughter cells. (**F**) Sometimes the prophage can be induced to become active. The prophage DNA is excised from the bacterial genome and enters the lytic cycle.

Bacteriophages are obligate parasites of bacteria, using the bacterial cell to replicate. Depending on their interactions with bacteria and their life cycle, they are divided into two types: lytic (virulent, productive) and lysogenic (temperate, dormant) (Figure 1). Certain bacteriophages have the ability to perform both lytic and lysogenic cycles (e.g., phage lambda of *Escherichia coli*). During the lytic cycle, a bacteriophage infects a target bacterium (live bacterial target cell), replicates therein, kills the bacterium by lysis, and releases multiple (20–200) [8] or hundreds to thousands of phages [9]. In detail, at the end of the lytic cycle, the phage protein (holin) makes pores within the cytoplasmic membrane and thus enables the phage-encoded endolysin (called ‘lysin’) to gain access and hydrolyze the peptidoglycan layer. This results in cell lysis and release of the progeny phages (produced by the host bacterium), which can infect other bacterial cells, thereby repeating the cycle [9]. The duration of the whole cycle may vary and is usually within 20–40 min to 1–2 h. Lytic phages have several potential applications.

In contrast, the lysogenic cycle does not result in the lysis of the host cell and progeny production. Instead, it leads to the integration of phage genetic material into the bacterial genome, and its, transmission into new cells (after cell division). The dormant phage is known as a prophage or endogenous phage (a latent form of phage). Under abnormal environmental conditions, the phage can become active and enter the lytic stage [10,11] (Figure 1).

Phages show specificity for various bacteria. Monovalent phages are specific to one type of bacterial species but polyvalent phages are able to attack different (two or more) bacterial species. Most works have indicated that bacteriophages targeting Gram-positive bacteria are not simultaneously effective against the Gram-negatives. Bacteriophages encode endolysins (phage lysins, hydrolases) which are involved in lysis of bacterial cell wall (peptidoglycan layer) from the inside at the end of replication (lytic cycle) thus resulting in release of the viral progeny phages. Moreover, bacteriophage endolysins can destroy the peptidoglycan layer when applied externally to the bacterial cell, and hence may play a role as novel antimicrobial agent. Exogenous activity of endolysins is particularly effective against Gram-positive bacteria, since they lack an outer membrane, unlike the Gram-negatives. Gram-negative bacteria are difficult to lyse because the outer membrane blocks access of endolysin to peptidoglycan. Endolysin-mediated external lysis of Gram-negative bacteria can be achieved; however through the use of a permeabilizing agent [12]. On the one hand, the selective ability of phages to attack certain bacteria allows for the selective elimination of pathogenic bacteria, but on the other hand restricts their use for therapeutic purposes.

**Figure 2:**
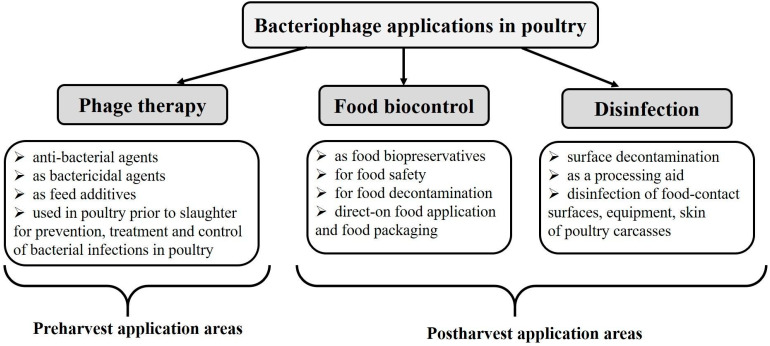
Examples of phage application in the poultry industry.

In the past decades, the emergence of multidrug antibiotic-resistant bacteria (MDR) has been reported as a result of too common and frequent use of antibiotics in human and veterinary medicine as well as in industry and agriculture. Moreover, MDR can be transmitted from food-producing animals to humans via direct contact between animals and humans, or through the food chain and the environment [13,14]. In the face of a loss of life-saving antibiotics and the lack of new ones, researchers have started the search for alternative means of fighting bacterial pathogens, such as vaccines, probiotics, prebiotics, bacteriophages, nanoparticles, antimicrobial peptides (AMPs), or anti-virulence compounds. There has been an explosion of research and interest in the usage of bacteriophages in the poultry veterinary medicine and the poultry industry. Thus, in turn, new companies developing bacteriophage preparations have appeared on the global market. Examples of phage applications in poultry farming, processing, and production are shown in Figure 2.

## 2. Phage Therapy of Bacterial Infections in Poultry

Public health concerns have increased the attention paid to pathogen, such as *Camylobacter jejuni (C. jejuni), Salmonella enterica* subspecies *enterica* serovar Enteritidis (*S.* Enteritidis)*, Salmonella enterica* subspecies *enterica* serovar Typhimurium (*S.* Typhimurium), *Eschericha coli (E. coli), Listeria monocytogenes (L. monocytogenes),* and methicillin-resistant *Staphylococcus aureus* (MRSA), because of the risk posed by poultry as a source of these pathogens. Most works have addressed the efficacy of bacteriophages in reducing bacterial count and in the control of bacterial infections in poultry, which are zoonotic and have a substantial impact on public health [15,16,17]. According to a recent report of the European Food Safety Authority (EFSA) and the European Centre for Disease Prevention and Control (ECDC) (2019), campylobacteriosis followed by salmonellosis, Shiga toxin-producing *E. coli* (STEC) infection, and yersiniosis were the most frequently reported zoonoses in the European Union (EU) [18]. Due to their restricted ability to kill bacteria, only the lytic bacteriophages are suitable for phage therapy applied to treat bacterial infections. Bacteriophages are much more specific than antibiotics are. It should be noted that antibiotic treatment not only kills the pathogenic bacteria but also affects the normal intestinal microbiota, potentially leading to dysbiosis, immunosuppression, and thus to secondary infections [14]. Hence, novel bacteriophage treatments, represent an excellent tool for the treatment of bacterial infections in poultry. Table 1 shows examples of commercial bacteriophage products.

### 2.1. Campylobacter

*Campylobacter* spp. bacteria are ubiquitous in various environments but prefer the gut of birds, which they colonize as commensals. Due to the conducive (optimal) body temperature, poultry has become a natural reservoir for *Campylobacter* species, representing the major source of human infections. *Campylobacter* spp. colonization in chickens takes place at poultry farms, approximately seven days after hatching. Although chickens are carriers of *Campylobacter,* they typically do not exhibit clinical signs or lesions. The prevalence of *Campylobacter* spp. in poultry flocks varies considerably from 2% to 100% [33]. Study results have indicated the high prevalence of *Campylobacter* in poultry at slaughter, where 100% of small intestinal samples and 91.5% of swabs from carcass surfaces were positive [34]. Other authors noted lower prevalence in broiler chickens (34.3%, cecum samples) [35]. According to EFSA and ECDC report, the prevalence of *Campylobacter* spp. was 71.6 % in turkeys and 26% in broilers in 2018 [18]. Two species have been reported to prevail in poultry: *C. jejuni* (86.1% or 64.6%) and *C. coli*. (13.9% or 35.4%) [34,35]. Similarly, at a farm level, the prevalence of *C. jejuni* (65.8%) was significantly higher than that of *C. coli* (12.6%) [36]. The persistent presence of *Campylobacter* contaminating the environment in slaughterhouses and poultry products has been reported worldwide. According to EFSA and ECDC, the highest prevalence of *Campylobacter* was observed in fresh meat from broilers (37.5%) followed by fresh meat from turkeys (28.2%) [18]. In addition, the increasing number of reports regarding *Campylobacter* antibiotic resistance (to fluoroquinolones, tetracycline, erythromycin, gentamicin) and virulence are moving forward the efforts to reduce *Campylobacter* [34,35,36]. Generally, the occurrence of bacteriophages specific to *Campylobacter* among commercial poultry or retail chicken products is low, and most of them belong to the family *Myoviridae*, rarely to *Siphoviridae* [35,37,38]. Recent experimental studies have provided evidence for the efficacy of phage treatment in reducing the *Campylobacter* colonization in chickens and thus in minimizing the risk of its entrance into the food chain. A phage cocktail containing virulent *Campylobacter* phages was used by oral route to treat broiler chickens colonized with *C. jejuni*. Bacteriophage predation of *C. jejuni* did not affect the microbiota but selectively reduced the abundance of *C. jejuni*. Authors have concluded that bacteriophage control to reduce *C. jejuni* levels in chickens could reduce human exposure and disease acquired through the consumption of contaminated poultry products [39]. Despite the obvious need for implementing novel solutions aimed to reduce the rate of infections induced by *Campylobacter* spp., there are still not available commercial phage products against these bacteria. This can be due to the fact that, compared with most other lytic phages, *Campylobacter* phages exhibit some characteristics which make their application rather difficult. The problems with the production of safe *Campylobacter* phage cocktails are associated mainly with the optimization methods for phages isolation, propagation and purification. Moreover, there are differences between *Campylobacter* phages within groups (i.a. in host range, lytic activity, kinetics), even they are genetically very similar, which make difficulties with appropriate selection of phage candidates for application. In addition, the emergence of phage resistant *Campylobacter* post-phage treatment have been reported (the frequency of 1–14%). Finally, the cost of production is also the next major consideration [39,40].

### 2.2. Salmonella

*Salmonella* is one of the main bacteria affecting commercial poultry and the second (after *Campylobacter*) of the most important zoonotic foodborne pathogens. *Salmonella* infections in poultry can be grouped into three categories: (1) Host-specific infections caused by nonmotile serotypes: *Salmonella enterica* subspecies *enterica* serovar Pullorum (*S.* Pullorum), *Salmonella enterica* subspecies *enterica* serovar Gallinarum (*S.* Gallinarum). *Salmonella* Pullorum causes pullorum disease (PD), an acute systemic disease of young birds. The infected adults are usually asymptomatic carriers. *Salmonella* Gallinarum causes fowl typhoid (FT) an acute or chronic septicemic disease that most often affects growing or mature birds. (2) Non-host-specific infections caused by motile serotypes referred as paratyphoid (PT) salmonellae: *Salmonella enterica* subspecies *enterica* serovar Enteritidis (*S.* Enteritidis), *Salmonella enterica* subspecies *enterica* serovar Typhimurium (*S.* Typhimurium). Other serotypes in poultry are i.a. *S.* Hadar, *S.* Infantis, *S.* Virchoff, *S.* Heidelberg, *S.* Kentucky, *S.* Anatum. Although PT infections occur frequently in poultry, they rarely cause acute systemic disease except in highly susceptible young birds under stressful conditions. Signs usually are seen only in young birds (less than four weeks of age). More often PT infections of poultry are characterized by asymptomatic and sometimes persistent colonization of the intestinal tract and internal organs, potentially leading to contamination of finished carcasses. (3) Avian arizonosis (AA) caused by *Salmonella enterica* subspecies *arizonae (S. arizonae*) is an acute or chronic disease of primarily young turkey poults. Adult birds generally show no clinical signs of disease but can be carriers [41].

Most human salmonellosis cases in the EU were induced by *S.* Enteritidis, and the proportion of human cases due this serovar was in 2018 at the same level as in 2018 as in 2017 (20.1 cases per 100,000 inhabitants). Similar to previous years, in 2018, *Salmonella* food-borne outbreaks were mostly caused by eggs and egg products. In food, the highest levels of *Salmonella*-positive samples were found in poultry meat. The EU flock prevalence of target *Salmonella* serovars in breeding hens, laying hens, broilers, and fattening turkeys decreased in recent years but stalled in breeding turkeys [18].

In the early 90s, Berchieri et al. [42] showed the efficacy of bacteriophages after simultaneous oral inoculation of chickens with phages and *Salmonella enterica* subspecies *enterica* serovar Typhimurium (*S.* Typhimurium). Bacteriophages reduced the viable numbers of *Salmonella* in the gut of chicken (crop, small intestine, cecum) during the experiment. Reductions of more than one log_10_ were seen in the crop and small intestine soon after inoculation and throughout the alimentary tract at three days post-inoculation. However, despite the phage multiplication, some phages (AB2) did not affect the number of *Salmonella* in cecum. No neutralizing antibodies to the phage were detected in the serum of chickens (at 32 dpi). Additionally, phages could be isolated from the in-contact birds.

Other reports confirmed that a bacteriophage cocktail composed of several phages was more effective in promoting the lysis of *Salmonella* spp. than a phage alone. In addition, the cocktail obtained from *Salmonella enterica* subsp. *enterica* serovar Enteritidis (*S.* Enteritidis), and serovar Typhimurium (*S.* Typhimurium), was also able to promote the lysis of other *Salmonella* serovars (Virchow, Hadar, Infantis). It seems that the significant reduction of *Salmonella* cell numbers in chicken cecum may be achieved after repeated treatment (oral re-administration) with the bacteriophage cocktail [43]. 

Other authors showed that bacteriophages might be effective alternatives to antibiotics to control fowl typhoid disease (*Salmonella* Gallinarum) in layer chickens. Chickens were orally fed with bacteriophages for seven days before the bacterial challenge and 21 days after the challenge. Bacterial re-isolation from the organs and mortality decreased significantly in the challenged chicken treated with the bacteriophages. The bacterial re-isolation rate and mortality were also lower in the contact chicken with phage treatment but not challenged with *Salmonella*. They were exposed to infection only by bacterial transmission from the closely housed challenged chickens. However, the bacterial re-isolation rate from the contact chickens without phage treatment was lower than from the challenged chicken [44].

Other studies reported the presence of *Salmonella*-specific bacteriophages in both sewage samples from poultry farms and infected broiler chickens. Bacteriophages from sewage water were administrated orally to chicks before oral *Salmonella* infections followed by 4 successive phage treatments after the bacterial challenge. No bacteria were detected in the cecum after the last (5th) dose, indicating that the chicks treated with phages were cured of *Salmonella* (at 15 dpi) [45].

Other authors have announced the complete genome sequence of bacteriophage isolated from water by using *Salmonella enterica* subspecies *enterica* serovar Enteritidis (*S.* Enteritidis). The bacteriophage can infect both *C. jejuni* and *S.* Enteritidis and can be used in phage therapy in poultry and to improve the biosafety of poultry meat [46].

Several bacteriophage products against *Salmonella* infections are available today [15,16]. In 2019, the first results were presented from the use of *Salmonella* phages at a large scale in a poultry production system [26]. The multiple administration of a bacteriophage mixture (SalmoFREE^®^) in drinking water was safe, and did not affect the behavior of chicken nor their production parameters. At the end of the cycle (day 33), *Salmonella* was reduced in cloacal swabs to 0%. Other product, Bafasal^®^ (Proteon Pharmaceuticals, Poland) is a feed additive intended for birds and administered with drinking water (Table 1). In trials and commercial usage, Bafasal^®^ has been shown to have a strong impact on food safety, reducing *Salmonella* levels as much as 200 times, while also improving feed conversion rate and reducing mortality. Bafasal^®^ has both a prophylactic and a post-infection interventional effect, while its administration does not require the waiting period for meat and eggs [19]. Another product, Biotector S1^®^ (CJ CheilJedangResearch Institute of Biotechnology, Seoul, South Korea), is the world’s first bacteriophage product, which can be used as a feed additive to control *Salmonella enterica* subspecies *enterica* serovar Pullorum (*S.* Pullorum), and *Salmonella enterica* subspecies *enterica* serovar Gallinarum (*S.* Gallinarum) in poultry (Table 1). The mortality rate recorded in three experimental groups of commercial broilers (5-week-old Ross), which received Biotector S1^®^ in different concentrations in feed (5 × 10^7^, 1 × 10^8^ and 2 × 10^8^ PFU/kg), decreased by 73% in comparison to the control group (11.81%) after the challenge. There were no significant differences in mortality between the experimental groups (2.78%, 3.13%, 3.13%). In the group of broiler breeders (67-week-old Ross) receiving bacteriophages (1 × 10^6^ PFU/kg), the mortality rate (45%) decreased by 53% when compared with the non-phage treated control (85%) after challenge. The highest drop of mortality (by 86%) was observed in layers (6-week-old Lohmann Brown) which received the same dose (1 × 10^6^ PFU/kg) before the challenge. The mortality in control group after challenge was 35%. In the Hy-Line Brown layers, also performance was improved in the phage treated group (1 × 10^8^ PFU/kg): 3% increase in egg production (trial 90.6%, control 87.5%), and 2.4% increase in egg mass (g/day/bird) (trial 59.2%, control 56.8%) [47].

### 2.3. Escherichia coli

*Escherichia coli* is a Gram-negative bacillus, a normal inhabitant of the digestive tract of birds, which is widely disseminated with feces. Most strains are nonpathogenic, however, certain pathogenic serotypes (avian pathogenic *Escherichia coli*—APEC) may induce disease, leading to mortality and condemnations. This opportunistic pathogen can act as both a primary and secondary pathogen. *E. coli*-associated infections are widely distributed among poultry of all ages and categories. Some strains, such as, enterohemorrhagic *E. coli* (EHEC), and its subgroup of Shiga toxin (Stx)-producing *E. coli* (STEC), are food-borne pathogens responsible for serious human diseases worldwide [48].

Bacteriophages infecting *E. coli* are called coliphages. The phage-based products intended for treatment colibacillosis in poultry are still not available on the market.

In a study of Barrow et al. [49], bacteriophage R, originally derived from human sewage, was effective in preventing and treating septicemia and cerebritis or meningitis in chickens. Chickens were inoculated intramuscularly or intracranially with *E. coli*, whereas phage preparations were administered by intramuscular injection (gastrocnemius muscle, right leg). An almost 100% mortality rate was noted in the non-treated 3-week-old and newly hatched chickens inoculated by both routes. Phages reached the brain in chicken, which were earlier intracranially infected with *E. coli.* They were able to multiply rapidly and decline the bacterial count. Moreover, the above authors demonstrated the ability of bacteriophage to protect chickens even when the preparation was administered (1–2 days) before being challenged with *E. coli* or during the onset of clinical signs. This may indicate that phages can persist long enough in the tissues and thus may be used in the treatment as well as in the prophylaxis of colibacillosis. Interestingly, the authors noted also that a commonly known virucidal disinfectant, Virkon^TM^ (Antec International, Sudbury, United Kingdom), was very effective against the phage used, however, these observations need to be confirmed in the future research.

Huff et al. [8] have demonstrated that aerosol spray of bacteriophages administered to 7-day-old chickens prior to the triple challenge with *E. coli* can prevent airsacculitis caused by *E. coli*. On the other hand, the aerosol spray of bacteriophages was ineffective when administered after the chicken had been challenged with *E. coli*. The effectiveness of treatment with bacteriophage seems to be dependent on the circulating bacteriophage titers. In opposition to the intramuscular injection of bacteriophages, the aerosol spray could have induced only low bacteriophage levels in the blood and only a few chickens had detectable levels. Further results demonstrated that bacteriophage treatment was comparable to enrofloxacin treatment. Moreover, when bacteriophage and enrofloxacin were used in combination, their synergistic effect improved the efficacy of colibacillosis treatments. Combining the antibiotic with bacteriophage therapy may reduce the levels of antibiotics used in treating bacterial diseases.

Other authors compared the efficacy of the antibiotic (chloramphenicol class) and oral phage therapy (from sewage) against enteropathogenic *E. coli* in 20-day-old chickens [50]. In the second week, no diarrhea was found in the birds receiving phages, while there were 12.4% diarrhea rates in the birds receiving antibiotic and 25.2% in the control group (treated with water). The death rate was 14.8% in the control group, which was 2-times and 5-times more than in the antibiotic and phage group, respectively. The authors concluded that phage was safe, non-toxic, and caused no disease in the chickens when compared with antibiotic treatments. Moreover, the chickens receiving phage had increased weight. The phage treatment had high specificity without affecting beneficial bacteria, which is very important for maintaining a favorable intestinal microecological homeostasis.

More recently, Tawakol et al. [51] showed that bacteriophage treatment (by intratracheal inoculation) reduced the severity and prevented the mortality of not only single APEC infection but also a mixed infection with APEC and infectious bronchitis virus (IBV). Additionally, in the mixed infection group, but not the single IBV infected group, the bacteriophage treatment significantly reduced the count of pathogenicity-shedding *E. coli* as well as IBV. 

### 2.4. Staphylococcus Aureus

*Staphylococcus aureus* is considered as the most common and pathogenic staphylococcal species isolated from poultry. It should be mentioned that staphylococci (including *S. aureus*) belong to the normal inhabitants of skin, mucous membranes of healthy birds and are ubiquitous in the poultry environment. Staphylococcal infections caused by *S. aureus* are a worldwide problem in chicken and turkey production and cause economic losses due to decreased production results as well as mortality and condemnation of carcasses at slaughter. Diseases caused by *S. aureus* infections include arthritis, synovitis, chondronecrosis, osteomyelitis, gangrenous dermatitis, subdermal abscesses (bumblefoot), green liver-osteomyelitis complex in turkey, and septicemia [52]. Some enterotoxin-producing strains can cause food poisoning in people. Poultry-associated food poisoning can occur due to the contamination of carcasses with *S. aureus* at processing (especially enterotoxin-producing strains). Methicillin-resistant *S. aureus* (MRSA) found in poultry meat may raise concerns as well [53].

The phage which attacks bacteria *Staphylococcus* bacteria is called a staphylophage. Based on the genome size, staphylophages were grouped into three classes: class I—*Podoviridae* (with the smallest genome), class II—*Siphoviridae* (intermediate genome size), and class III—*Myoviridae* (the largest genome) [54]. Bacteriophages induced from *S. aureus* strains originating from broiler chickens and turkeys belonged to the family *Siphoviridae* of the order *Caudovirales*, and had an icosahedral head and a long, thin, non-contractile flexible tail, and double-stranded DNA. They belonged to 3 serogroups (A, B, and F with Fa, Fb subgroups) and exhibited strong lytic properties against *Staphylococcus* strains but also against other bacteria. Although bacteriophages showed strong specificity to *S. aureus*, some of them harbored the enterotoxigenic genes, which makes them useless in phage therapy [55]. From the therapeutic point of view, *Myoviruses* are considered the most interesting staphylococcal phages. Although podoviruses are strictly lytic they are very rare [54]. Today, there are no phage preparations intended for the prophylaxis and treatment of *S. aureus*-induced infections in poultry.

### 2.5. Clostridium

*Clostridium perfringens* is a Gram-positive anaerobic spore-forming non-motile rod-shaped bacterium. It is widespread in the natural environment and a normal inhabitant of the intestinal microbiota of poultry. At low population levels (<10^4^ CFU), it is non-pathogenic, however, its pathogenicity is mainly associated with toxins. In poultry, *Clostridium perfringens* type A and type C producing toxins can induce necrotic enteritis (NE) which in its both forms (acute clinical and subclinical) represents one of the most economically significant poultry diseases. Moreover, enterotoxin-positive *C. perfringens* can cause foodborne diseases, whereas the infected poultry meat may be a source for human intoxications [56]. 

Bacteriophages induced from *C. perfringens* strain originating from poultry intestines, soil, sewage, and poultry processing drainage water were identified as members of the order *Caudovirales* in the family *Siphoviridae and Podoviridae*. Many strains of *C. perfringens* remained resistant to the phages. Additionally, the activity of phages seemed to be restricted to a specific isolate of this bacterium [57,58]. Some authors suggested that endolysin encoded by phages of *C. perfringens* may be especially useful for control of this bacterium. The results indicated that endolysin may be active against all tested strains of *C. perfringens*, although variation in the sensitivities of different strains is possible [59,60,61].

Miller et al. [62] reported the efficacy of bacteriophage (INT-401) in controlling necrotic enteritis (NE) caused by *C. perfringens* in broiler chickens. The phage treatment via drinking water or feed allowed not only to reduce the mortality rate but also to improve weight gain and feed conversion ratio (FCR) in the experimentally infected broiler chicken.

Other authors evaluated the combined effect of the phages and bacteriocin against *C. perfringens* [63]. They observed the synergetic effect of two lytic phages P4, A3 (10^8^ PFU/mL) and a heat-, pH-stable bacteriocin of *S. hyointestinalis* against *C. perfringens* isolated from chickens and pigs. The combined treatments with phages and bacteriocin have significantly reduced the bacterial population (by 6.20 log units) than treatment with the phage (by 1.36 log units, phage P4; 4.41 log units, phage A3) or bacteriocin alone (by 3.8 log units). The treatment with phage or bacteriocin alone resulted in instant bactericidal and bacteriostatic effects against *C. perfringens*, however, regeneration of *C. perfringens* was also observed. As the data revealed, combining the phages with bacteriocin may represent potential options for the control of *C. perfringens*.

## 3. Reduction of Food Contaminations (Biocontrol)

Decontamination is a complex problem involving the removal and neutralization (inactivation) of microorganisms in food products, which enables retarding their putrefactive and aging processes. Counteracting human infections involves mainly the elimination of bacteria from foods. Many methods are used to help improve the safety of foods, however, not all are appropriate for fresh meats and products, and their usage is limited, e.g., heat pasteurization, high pressure, radiation. Other methods, such as the use of organic acids (due to their low pH), are effective, however the bactericidal activity to be induced requires high concentrations of acids which may have an impact on meat quality and visual appearance (texture, color, oxidative stability, pH, etc.) [15]. The concept of reducing pathogens in food by using phages has mainly addressed raw and raw meat and ready-to eat (RTE) products, and also decontamination of carcasses [64]. Nowadays, there are some commercialized phage products against *Listeria monocytogenes*, *Salmonella*, *Shigella*, and *E. coli* that use phages as food biopreservation agents (Table 1).

*Listeria monocytogenes* poses a particular foodborne hazard because of the ability to grow and replicate at refrigeration temperatures. There has been an increasing trend of confirmed human listeriosis cases from 2014 to 2018 [18]. The United States Food and Drug Administration (U.S. FDA) and the U.S. Department of Agriculture (USDA) approved ListShield^TM^ (formerly LMP-102, Intralytix Inc.Baltimore, USA) as a food additive for ready-to-eat meat and poultry products. ListShield^TM^ is a mixture of six lytic bacteriophages that specifically target *L. monocytogenes* and can be applied directly on food. The manufacturer has claimed that ListShield™ does not affect the organoleptic quality of foods [29].

Another known bacteriophage-based product against *L. monocytogenes* called Listex^TM^ contains a single phage P100 at a concentration of 2x10^11^ PFU/mL. Bacteriophage P100 has been shown not to pose a health risk and to be non-toxic. Listex^TM^ P100 received the GRAS (generally recognized as safe) status issued by the FDA in 2006 and was intended for raw and RTE foods at levels not to exceed 10^9^ PFU/g, usually as a spraying or dipping suspension. EFSA issued a scientific opinion on Listex^TM^ P100 and recommended undertaking more studies on its efficacy in naturally contaminated RTE foods [31,32].

According to Bigot et al. [65], the effectiveness of the phage treatment depends on the initial concentration of phages. In their experiment, *L. monocytogenes* and then phage stock suspension (FWLLm1) were applied in similar volumes onto the surface of the commercially available vacuum-packed RTE chicken breast roll. Chicken samples were then vacuum-packaged and incubated at 30 and 5 °C. The number of *L. monocytogenes* decreased on a RTE vacuum-packed chicken samples tested at 5 °C. However, at 30 °C there was an immediate reduction in *Listeria* concentration but then re-growth occurred. This re-growth was prevented over 21 days of incubation. After 21 days, *Listeria* cells that had survived in the phage-treated samples may have commenced the re-growth. It should be underlined that the temperature of 30 °C and duration of incubation were abnormal and deviant from typical food storage conditions. Moreover, the authors concluded that the rate of phage and host application should be reported per unit area instead of per unit weight (with an unspecified area being inoculated).

The most recent studies by Kim et al. [66] revealed the efficacy of the bacteriophage cocktail in reducing *Salmonella enterica* subspecies *enterica* serovar Enteritidis (*S.* Enteritidis) in raw chicken breast meat at refrigeration temperatures (4 °C). These four lytic phages were isolated from a river proximate to a duck farm. All belonged to the *Myoviridae* and *Siphoviridae* family and had a broad lytic effect on 96% (24/25) of different serovars of *Salmonella enterica* subsp. *enterica* (*S.* Enteritidis, *S.* Typhimurium, *S.* Paratyphi A, *S.* San Diego, *S.* Typhi). Other studies showed the efficacy of a phage-based product called SalmoFresh^TM^ (Intralytix Inc., USA) in reducing *Salmonella* on chicken breast fillets stored under aerobic and also modified atmosphere conditions (95% CO_2_/5% O_2_) [67].

Even the newest methods for food decontamination will, however, not replace the standard hygienic principles. Nevertheless, they offer solutions that would allow for taking greater care over food safety and for extending the shelf life of food products, which in turn can contribute to lesser food waste.

## 4. Use of Bacteriophages as Disinfectants

Various approaches have been proposed to reduce the numbers of bacteria associated with poultry facilities, e.g., legal regulations, strict biosecurity strategies, and specific conditions. Since the main source of poultry meat contamination was confirmed to be the flock, it is reasonable to suggest that bacteria-free meat could be achieved by reducing their prevalence at a farm level. In addition, an aerosol spray of poultry and litter in production facilities may help to prevent horizontal transmission of the pathogen. Bacteriophage-based products may be used as biosanitizers in hatcheries, farms, transport crates, poultry processing plants, and food contact surfaces. Moreover, bacteriophages, have been considered as effective in inhibiting biofilm formation and dispersion of mature biofilms produced by pathogenic bacteria on surfaces commonly found in the poultry industry. The material that the equipment elements are made of is also important. It has been shown that glass and stainless-steel materials significantly promoted biofilm formation by *Salmonella* spp. compared to polyvinyl chloride surface (PVC). Moreover, *S.* Enteritidis and *S.* Heidelberg had stronger biofilm-forming ability than other serovars tested [68].

The phage-based surface disinfectants, such as BacWash^TM^ (OmniLytics Inc., USA), target *Salmonella* and can be applied as a wash, mist, or spray, and used directly on the live animals prior to slaughter. Similarly, Ecolicide PX™ (Intralytix) *E. coli* O157:H7 has been developed for the decontamination of the skin of live animals prior to slaughter [21]. Atterbury et al. [69] reported the efficacy of bacteriophage in reducing the number of recoverable *Campylobacter jejuni* cells on artificially contaminated skin of broiler chicken. Other authors assessed five phages from chicken feces, characterized and selected to be used as biosanitizer [70]. They compared the activity of a phage cocktail with chemical agents in reducing *Salmonella* Enteritidis on chicken skin. Skin samples were dipped in 100 mL of the phage cocktail at 10^9^ PFU/mL for 30 min and experimentally contaminated with 1 × 10^5^ CFU/cm^2^ *Salmonella*. Authors have demonstrated that bacteriophages reduced *S.* Enteritidis loads on chicken skin at refrigeration temperature and short contact time. A similar level of *Salmonella* reduction (by an average of 1 log CFU/cm^2^) was also obtained after using chemical agents.

El-Gohary et al. [71] demonstrated that the treatment of litter by spraying with a bacteriophage preparation targeting *E. coli* was a practical and efficacious tool to prevent colibacillosis in broiler chickens resulting from exposure to *E. coli* in the environment.

## 5. Threats Arising from the Use of Bacteriophages in Poultry

Each phage-based preparation intended for use in veterinary medicine of poultry, in poultry production, and poultry industry should, most of all, be safe and effective. Dosage and delivery route (including the preparation of standardized formulations), administration timing of phage-based products, as well as the concomitant use of other preparations (e.g., competitive exclusion) or vaccinations are also of the utmost significance. The persistence of bacteriophages in/on food may vary with each bacteriophage, and with the conditions of application (e.g., dose) and environmental factors (e.g., temperature). The refrigeration temperatures may enhance the persistence of bacteriophages on the surface of meat products [72]. 

The approval for the use of bacteriophages as feed additives or directly on RTE food has elicited controversies and discussions. Even though ample research on bacteriophage applications has provided many positive conclusions, there are still some disadvantages and unknowns [73]. Additionally, the narrow range of activity of phages might also be problematic in disease control and may restrict the number of types of infections for which such an approach may be appropriate [49]. 

Due to the specific ability to kill bacteria, only strong lytic phages with known nucleotide sequences should be used. Lysogenic phages incorporate their genetic material into the bacterial genome. Consequently, they may act as vehicles for horizontal gene transmission between bacteria, or animals or to humans via the food chain. Due to the advance in research, it seems feasible to understand the gene flow between phages and their hosts. Hence, it would also be possible to avoid potentially adverse bacteriophages or to re-design them into the ones that would be incapable of transferring undesirable traits or any other gene dissemination systems. Many authors used different phages in the same product, enlarging the lytic spectrum and delaying the occurrence of resistance to phages. It seems that a phage cocktail is required for effective phage therapy [17].

The efficacy of bacteriophages depends on, among others, their good adaptation to replication and survival in required conditions. To achieve their optimal efficacy, it seems advisable to improve methods of phage selection and their isolation from the host environment [64]. It is essential to find better systems for phage purification ensuring the appropriate removal of elements of bacterial origin. Phages should be apyrogenic and non-allergenic (sterility test, lack of residual endotoxin). 

It is generally known that bacteriophages are immunogenic and able to elicit specific antibody humoral responses which might influence phage therapy in humans and animals including poultry [74,75,76,77,78]. Although no anti-phage antibodies were found in the first safety oral trial in humans [79], later studies indicated that phage therapy may induce various levels of antibodies which may not necessarily affect the outcome of therapy [80]. Other results suggest that the antiphage activity in human sera depends on the route of phage administration and phage type [76]. The phage-antibody interactions do not necessarily lead to phage inactivation. However, the various phages might differently respond to neutralization by antibodies. Interactions between phage and host’s immune system are not well known. Some authors have recommended phages screening for their ability to avoid antibody neutralization [81]. Modern technological systems (e.g., phage encapsulation) have been developed to increase phage activity in treating intracellular infections and phage safety of those which are directly added to food products and animal feed [82].

The importance of the phage concentration applied has been also shown in the studies on phage therapy in chickens (>10^10^ PFU/mL) and on RTE poultry products (10^7^ PFU/cm^2^). The presented data demonstrate that only high concentrations of phages are effective in ensuring a significant reduction in mortality and in foodborne pathogens [42,65]. On the other hand, the high doses and very long time of exposure may induce neutralizing antibodies. Moreover, the phages can accumulate in different organs. Żaczek et al. [80] found that phage dose, not the level of purification of phage preparation, plays an important role in immunogenicity of therapeutic phage preparations in humans. According to the literature phage formulations containing 10^5^ to 10^11^ PFU /dose have been administered orally to humans [74]. It should be noted that most phages can be destroyed when exposed to low pH of the stomach. Currently, several GRAS approvals permit the application of phage preparation up to 10^8^ PFU/g of food [15,22,23].

Many phage-based preparations have been commercialized and licensed for use in the United States, however, their implementation in the EU required more regulations and scientific opinions. Each phage should be assessed on a case-by-case basis for the nucleic acid sequence to demonstrate the impossibility of a lysogenic cycle and the absence of any potential virulence factors and/or antimicrobial resistance genes. The EFSA encouraged the research into specific bacteriophage-pathogen-food combinations to assess the issue of bacteriophage persistence in foods, and their ability to prevent recontamination with the bacterial pathogen [72].

Currently, there are no regulations or control methods aimed at monitoring bacteriophages and their consumption by humans [4]. Susceptibility of the bacteria to the phage as well as phage stability and efficiency should be monitored during the phage treatment. Moreover, scientific efforts should be undertaken to develop methods for preventing the spreading of phage resistant bacteria. 

It seems that one of the challenges may be an economic aspect associated with phage large-scale production to cover the possible needs of the poultry market. Most recently, Torres-Acosta et al. [27] developed a bioprocess model and performed an economic analysis of different production scenarios for the scaled-up generation bacteriophage cocktail intended for the poultry industry. The best cost-effective results could be obtained when one bioreactor (156 liters) was used for six phages, then a 0.45 μm filtration for removal of biomass, and a 0.22 μm filtration for sterility. Based on experimental-theoretical data, the applied configuration could supply 210 million chickens and give 0.02 USD production costs per chicken. Results indicate that the production titer has a crucial impact on the subsequent decrease in production costs; therefore, this parameter which requires proper optimization and improvement.

## 6. Conclusions

Despite skepticism in bacteriophages application in the poultry industry, many studies indicated their efficacy and considered them to be a useful alternative to antibiotics in the age of multidrug resistance and in the growing tendency to moving towards the post-antibiotic era. Future investigations could focus on the specific phage-bacterium interactions, pharmacodynamics, and mechanisms of coevolution between phages and bacteria.

## Figures and Tables

**Table 1:** Bacteriophage products addressed to the poultry industry.

Target Bacteria	Product Name	Manufacturer	Bacteriophages	Notes	Ref.
*Salmonella*	Bafasal^®^	Proteon Pharmaceuticals (Łódź, Poland)	3 phages: 3ent1, 8sent65 and 8sent1748, mixed in equal concentration	regulatory-approved feed additive for use in poultry to eliminate or prevent *Salmonella* infection in the digestive tract in poultry can be added to drinking water	[19,20]
*Salmonella* Gallinarum, *Salmonella* Pullorum	Biotector^®^ S	CJ CheilJedang Research Institute of Biotechnology (Seoul, South Korea)	nd	can be applied on animal feed to control *Salmonella* in poultry	[21]
*Salmonella enterica*	SalmoFresh™	Intralytix Inc. (Columbia, MD, USA)	6 lytic phages	for treating food FDA-approved, granted GRAS status	[15,21]
*Salmonella enterica*	SalmoPro^®^	Phagelux (Montreal, QC, Canada)	2 phages: BP-63, BP-12	for use as an antimicrobial processing aid to control *Salmonella* on food, when applied onto food surfaces up to l0^8^ PFU/g of food FDA-approved, granted GRAS status	[15,21,22]
*Salmonella*	Salmonelex™ (PhageGuard)	Micreos Food Safety BV (The Netherlands)	2 phages	for use as an antimicrobial on foodstuffs to control *Salmonella* at an application rate of up to 10^8^ PFU/g of food can be sprayed topically or added to chill tank water FDA-approved, granted GRAS status	[23]
*Salmonella*	PhageGuard S^TM^	Micreos Food Safety BV (Wageningen, The Netherlands)	2 phages: Fo1a and S16	water-based phage solution can be applied by spraying, dipping, immersion targeted to eliminate *Salmonella* in food products can be applied on fresh poultry or meat pre-grinding or pre-packaging for decontamination of surfaces FDA-approved, granted GRAS status	[15,24]
*Salmonella*	BacWash^TM^	OmniLytics Inc. (Sandy, UT, USA)	nd	for disinfection of surfaces	[25]
*Salmonella*	SalmoFREE^®^	Sciphage (Bogotá, Colombia)	6 lytic phages	for therapy and control *Salmonella* in poultry farm	[26,27]
*Escherichia coli* O157:H7	EcoShield^TM^	Intralytix Inc. (Columbia, MD, USA)	3 lytic phages: ECML-4, ECML-117, ECML-134 in the *Myoviridae* family isolated from the environment 10^10^ PFU/mL in PBS, pH 7.4	for treating food FDA-approved, granted GRAS status	[15,28]
*Escherichia coli* *O157:H7*	Ecolicide PX™	Intralytix Inc. (Columbia, MD, USA)	nd	for pre-harvest interventions applied on hides of live animals for reducing contamination prior to animal entering processing facility	[21]
*Listeria monocytogenes*	ListShield^TM^	Intralytix Inc. (Columbia, MD, USA)	6 phages: LIST-36, LMSP-25, LMTA-34, LMTA-57, LMTA-94, LMTA-148	food biopreservative for treating food for use as food processing aid FDA-approved, granted GRAS status	[15,28,29]
*Listeria monocytogenes*	Listex™ P100 (PhageGuard)	Micreos Food Safety BV. (Wageningen The Netherlands)	Phage P100	applied by spraying or dipping food biopreservative to prevent *Listeria* contamination on food products and food processing facilities. can be applied on RTE meat FDA-approved, granted GRAS status	[15,25,30,31,32]

FDA (U.S. Food and Drug Administration); GRAS (generally recognized as safe); PFU (plaque forming units); RTE (ready-to-eat); nd (no data).
